# Removal of Cu^2+^, Fe^2+^ and SO_4_^2−^ ions from industrial wastewater by ion exchange resins contained in a rotating perforated cylindrical basket of different heights

**DOI:** 10.1038/s41598-023-29956-4

**Published:** 2023-02-24

**Authors:** K. K. Zakaria, H. A. Farag, D. A. El-Gayar

**Affiliations:** Chemical Engineering Department, Faculty of Engineering, Alexandria University, Alexandria, Egypt

**Keywords:** Environmental sciences, Engineering

## Abstract

The present study is concerned with the development of a new cylindrical basket filled with ion exchange resin. The performance of the reactor was examined by removing Cu^2+^, Fe^2+^ and SO_4_^2−^ ions from synthetic wastewater. Variables studied were the initial ion concentration in the solution, contact time, resin height inside the cylindrical basket and cylindrical basket rotational speed. Dimensionless analysis was used to obtain a mass transfer correlation for each of the mentioned ions suitable for scale up and design of the present reactor. The experimental results revealed that both the percentage and the rate of removal of (Cu^2+^, Fe^2+^ and SO_4_^2−^) ions decrease as the initial ion concentration in the solution increases, while they increase as the contact time, rotational speed and (L/d) ratio increase. Both Langmuir’s and Freundlich’s adsorption isotherms were examined and it was found that Langmuir’s adsorption isotherm gives a better fitting for the obtained data than Freundlich’s. Regeneration ability was tested, which revealed the high resin efficiency upon operating several consequence cycles that could reach 4 cycles with a slight decrease in the removal efficiency.

## Introduction

Many industrial activities generate a significant amount of heavy metal ions in water via their wastewater effluents, which negatively impact the environment, aquatic life, and human health^1^. Social pressure for a safe environment has forced governments to legislate more stringent measures for wastewater treatment, especially for non-biodegradable pollutants such as heavy metals, to avoid contamination of the water bodies (rivers, oceans, and seas). According to this policy, the present work is concerned with (Cu^2+^, Fe^2+^ and SO_4_^2−^) removal from wastewater. Copper and iron ions are found with variable concentrations in several industries, such as the metal finishing industry, the printed circuit industry, and the effluent of the mining industry.

An example of the simultaneous presence of (Cu^2+^, Fe^2+^ and SO_4_^2−^) in the wastewater of copper plating plants where steel is coated with copper from a CuSO_4_ bath. The rinsing solution contains Cu^2+^and SO_4_^2−^ ions which should be removed to recycle water. Fe^2+^, SO_4_^2−^ comes also from the acid pickling step of the steel work piece in H_2_SO_4_ to remove oxides prior to plating.

Copper and iron ions in wastewater, even with low concentrations, could be harmful and cause serious diseases like liver damage in the worst case. Copper discharge to surface water could be of great concern as it will have the form of a suspended solid (sludge) or even as a free ion starts with small traces and builds up till it exceeds a relatively high concentration of which soil and most attached plants won’t be able to survive, moreover animals on lands polluted with copper would be poisoned as the worst case^2^. The presence of iron is essential for the biological system due to its vital role in the synthesis of hemoglobin, however higher dose of iron is considered to be a serious poisonous to newborn babies and even humans in general as the digestive system absorbs the iron content from water which will cause the failure of its cells, iron also cause kidney and liver damage as well as a malfunction in the cardiovascular system^3^. Knowing that the maximum allowable limit of Cu^2+^ and Fe^2+^ in water bodies is 1 and 0.3 mg/L respectively according to EPA^4,5^. The presence of sulfate ions in water, either surface or groundwater, is of high concern to many environmental, industrial, and health institutions because the high content of sulfate ions in water could favor the formation reaction of hydrogen sulfide, which is harmful to both aquatic life and also industrial equipment. Sulfate ions in water could be sourced from acid mine drainage and also from other several industries, high content of sulfate ions moving through metal pipes such as Mn, Ni, Cu...etc. could cause the release of these metals into the water causing a direct threat to aquatic life and human health^6^. High SO_4_^2−^ concentrations in water bodies above the allowable limit (250 mg/L) can cause several bad effects on humans and animals according to EPA^7^. Besides, SO_4_^2−^ can be reduced by anaerobic sulphate reducing bacteria to sulphide ions which is highly lethal and corrosive to metallic structures^8^.Sulfates are discharged from several industries such as textiles, dyes, fertilizers, metal, and plating industries. Several different methods for the removal of heavy metal ions and other pollutants from wastewater have been studied over the past decades, such as chemical precipitation, electrodeposition, membrane filtration, liquid–liquid extraction, ion exchange process, electrocoagulation etc. The ion exchange process was found to be the most suitable method for wastewater containing low to moderate amounts of heavy metal ions due to its high removal efficiency in addition to its reasonable cost^9^. Several types of adsorbents have been tested for the removal of heavy metal ions over the past decades like activated carbon, zeolites (organic resin), and synthetic resin, there are several types of synthetic resin used for different removal purposes like strong acidic cation exchange resin with sulfonic acid as a functional group, strong base anion exchange resin with the quaternary amino group as a functional group, weak acidic cation exchange resin with carboxylic acid as a functional group, and weak base anion exchange resin with primary, secondary and/or ternary amino group as a functional group^10,14^ Ion removal by ion exchange resin involves two steps, namely (1) mass transfer from the bulk solution to the resin material. (2) ion exchange between the removable ion and the exchanger resin^15^. The aim of the present work is to develop a new ion exchange reactor made of a rotating cylindrical perforated basket, filled with ion exchange resins. The rate of removal of Cu^2+^, Fe^2+^ and SO_4_^2−^ from synthetic wastewater was studied by the suggested reactor. Variables studied were ion concentration, the effect of resin height inside the basket to the basket diameter ratio (L/d), and the rotation speed of the basket. Previous studies proved that using either fluidized bed (Agitation of resin freely in solution) reactor or fixed bed reactor has drawbacks, where mass transfer of a fluidized bed is low because of low slip (relative) velocity between the resin particles and the solution while fixed bed suffers from high-pressure drop and hence high pumping power and high operating costs of the process^9,16,17^. The present study seeks to reach a dimensionless mass transfer correlation that can be used in practice for the design and scale up of the present reactor.

## Experimental part

### Resin and pretreatment

A strong-acid cation (SAC) exchange resin TRILITE MC-10H was used for the removal of (Cu^2+^, Fe^2+^), while a strong-base anion (SBA) exchange resin TRILITE MA-12 was used for the removal of (SO_4_^2−^). Table 1 shows the physical, and chemical properties and pretreatment of the resin^18^.

**Table 1:** Physical, chemical properties and pretreatment of the used resin.

Properties	TRILITE MC-10H	TRILITE MA-12
Grade	Industrial grade	Industrial grade
Type	Strong acid cation	Strong base anion
Functional group	Sulfonic acid	Type 1 Quarternary amine
Physical form	Khaki translucent spherical beads	Beige translucent spherical beads
Ionic form	H^+^	Cl^-^
Matrix	Styrene-DVB, Gel	Styrene-DVB, Gel
Bulk density (g/L)	800	680
Particle size (µm)	660 ± 50	580 ±50
Total capacity (eq/L)	2	1.3
Pretreatment process for TRILITE MC-10H		
Immersing the resin in 1 M NaOH solution for 45 min (To make sure that all the resin is in the Na^+^ form)		
Washing the resin several times with distilled water (To remove any excess of OH^-^) Immersing the resin in 1 M HCl solution for 45 min (To replace all Na^+^ with H^+^)		
Washing the resin several times with distilled water		
Drying the resin at room temperature for 1 day		
Drying the resin in a drying oven at 100–110 °C for 2 h		
Pretreatment process for TRILITE MA-12		
Immersing the resin in 1 M NaOH solution for 45 min (To make sure that all the resin is in the OH^-^ form)		
Washing the resin several times with distilled water (To remove any excess of Na^+^)		
Drying the resin at room temperature for 1 day		
Drying the resin in a drying oven at 100–110 °C for 2 h		

### Apparatus

**Figure 1:**
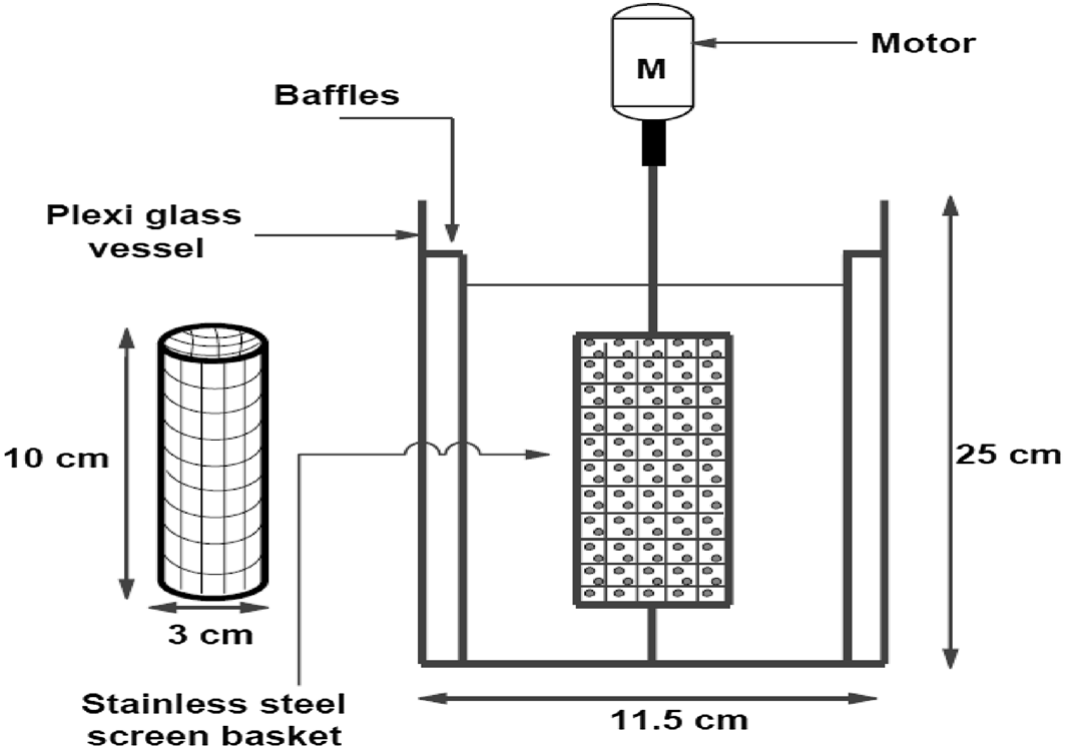
schematic diagram for the experimental setup.

The experimental setup shown in Fig. 1 describes the present work which consists of a plexiglass vessel of 2.5 L with 4 baffles fixed on the inside wall of the vessel, a stainless-steel screen basket of mesh no.40 with a top removable cover for ease of resin packing and unpacking, where the basket is connected to a rotating shaft attached to an electric motor with a variable speed controller.

### Procedures

- Prior to each run, both the plexiglass vessel and the stainless-steel basket were washed with distilled water and dried.
- 2 L of freshly prepared required solution with the required concentration of either copper, iron or sulfate was added into the vessel.
- The required amount of previously treated resin (either cation or anion) was added into the stainless-steel basket, and then the top of the basket was closed tightly with a stainless-steel cover of the same basket material to avoid resin escape during basket rotation.
- The stainless-steel basket shaft was connected to the motor and fully immersed in the solution inside the vessel.
- Adjustment of the motor speed at the required value was carried out by a variac; an optical tachometer was used to measure the rotation speed.
- Withdrawal of a 10 ml sample from the vessel (from the same point) every 10 min for Cu^2+^ ions removal and every 5 min for Fe^2+^, SO_4_^2−^ ions removal.
- Copper ion samples were analyzed using iodometric titration while iron ion samples were analyzed using redox titration, while sulfate ion samples were analyzed using “Hach DR3900” spectrophotometer^19^.
- All runs were carried out under a constant temperature of 25 ± 1 °C and pH 5. Solution viscosity and density were obtained using a viscometer and a density bottle, respectively; then the diffusion coefficient was obtained from literature at 25 °C^20,21^.

### Studied parameters

- Initial (Cu^2+^, Fe^2+^, SO_4_^2−^) ion concentration (200,300,400 mg/L)
- Cylindrical basket rotational speed (200,300,400,500,600,700 rpm)
- Ratio between resin height inside the cylindrical basket and the diameter of the cylindrical basket (0.59, 0.47, 0.35) for strong cation resin and (0.69, 0.55, 0.41) for strong anion resin.
- Regeneration ability of the strong cation resin

## Results and discussion

### Mass transfer analysis & data correlation

According to previous studies, the rate-determining step of the ion exchange process is either the film diffusion or the intra-particle diffusion step^16^. The former is due to the resistance resulting from the boundary layer around the ion exchange resin, while the latter is due to the resistance resulting from the porosity of the resin particle. Agitation affects the film diffusion step; conversely, the particle diffusion step is not sensitive to agitation. To determine the rate of the (Cu^2+^, Fe^2+^, SO_4_^2−^) ion removal via the ion exchange process using a batch reactor, the following equation was used, assuming a pseudo-first order reaction^22^.

1 $$- V\frac{dc}{{dt}} = KAC$$

By integration at t = 0, C = C_0_ and t = t, C=C the equation becomes

2 $$Ln \frac{{C_{0} }}{C} = \frac{KA}{V} t$$

where C_0_: initial ion concentration (mg/L), C: ion concentration at a given time (mg/L), V solution volume (cm^3^), t: time of reaction (s), K: rate constant (cm/s), A: resin particle surface area (cm^2^).

3 $$A = \frac{{6W}}{{d_{p} \uprho _{r} }}$$

where W: resin mass (g), d_p_: resin particle average diameter (cm), ρ_r_: resin density (g/cm^3^).

**Figure 2:**
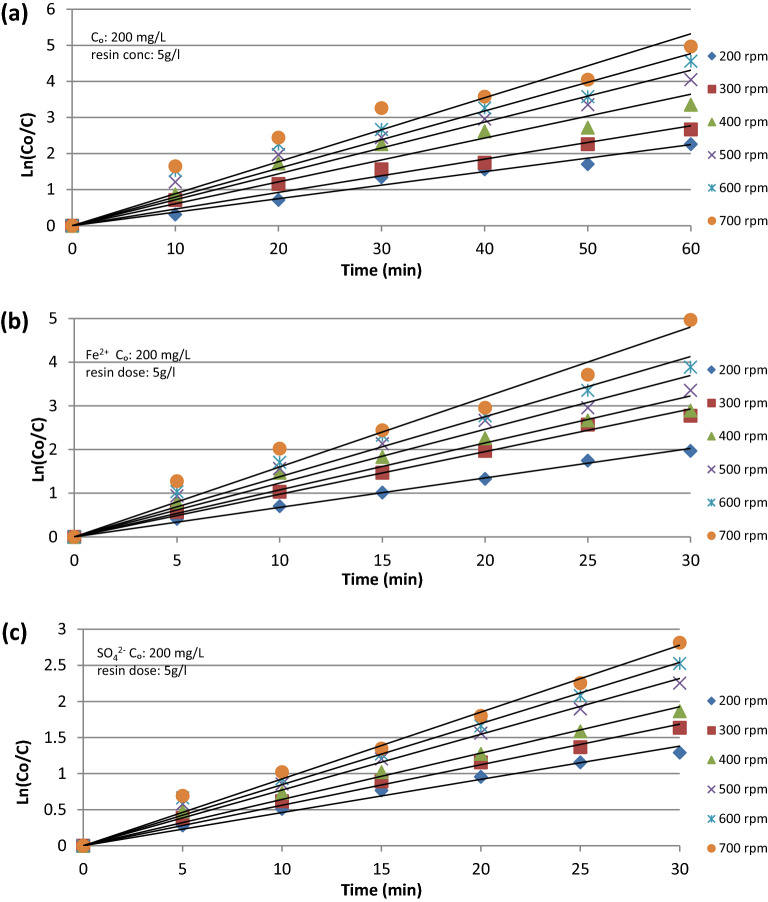
Relation between C0/C and time at different basket rotational speed for (**a**) Cu^2+^ ion, (**b**) Fe^2+^ ion, (**c**) SO_4_^2−^ ion.

The data presented in Fig. 2a–c show a typical plot of Ln (C_0_/C) versus time at different rotational speeds, the data fit Eq. (2) which emphasizes that the reaction is a first-order diffusion reaction. From Fig. 2a–c which represents Eq. (2), the slope of each straight line is (KA/V), from which the (Cu^2+^, Fe^2+^, SO_4_^2−^) removal mass transfer coefficients were calculated under different operating parameters.

**Figure 3:**
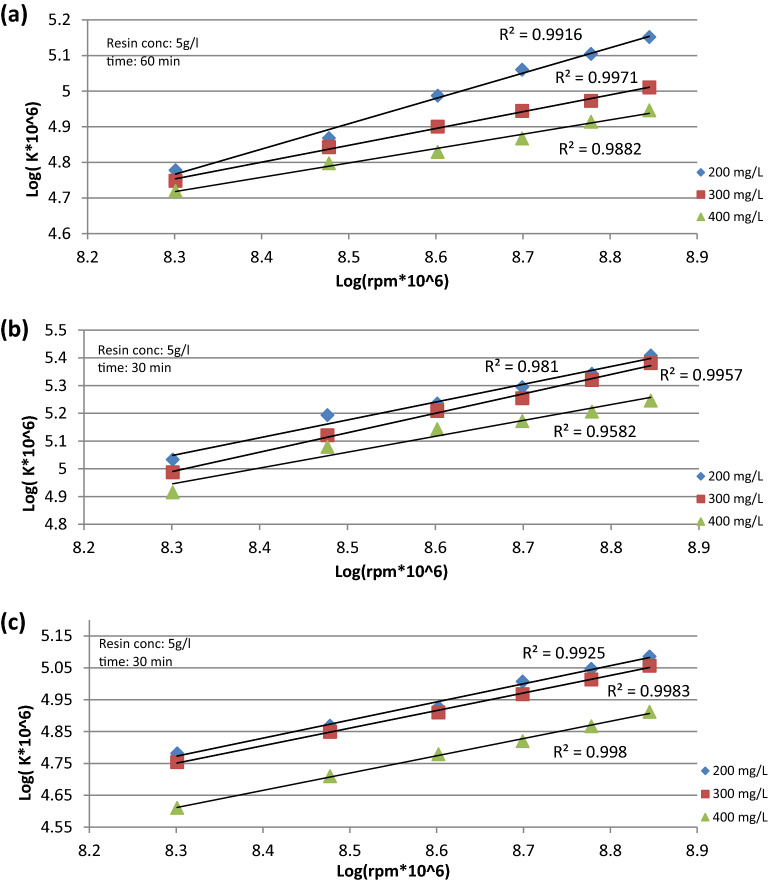
Relation between K and rpm at different initial ion concentration for (**a**) Cu^2+^ ion, (**b**) Fe^2+^ ion, (**c**) SO_4_^2−^ ion.

The data presented in Fig. 3a–c show the relation between rotational speed and the mass transfer coefficient, as the cylinder rotation speed increases, the mass transfer coefficient (K) increases. According to the equations:

4 $${\text{For}}\;{\text{Cu}}^{2 + } \quad \quad K = \alpha N^{0.528}$$

5 $${\text{For}}\;{\text{Fe}}^{2 + } \quad \quad K = \alpha N^{0.6388}$$

6 $${\text{For}}\;SO_{4}^{2 - } \quad \quad K = \alpha N^{0.553}$$

The exponent 0.528 from Eq. (4) is in agreement with the value of 0.5 obtained by El Shazly et al. (for his rate of mass transfer study at the wall of a rectangular agitated vessel with a 90° pitched blade turbine)^23^.

**Figure 4:**
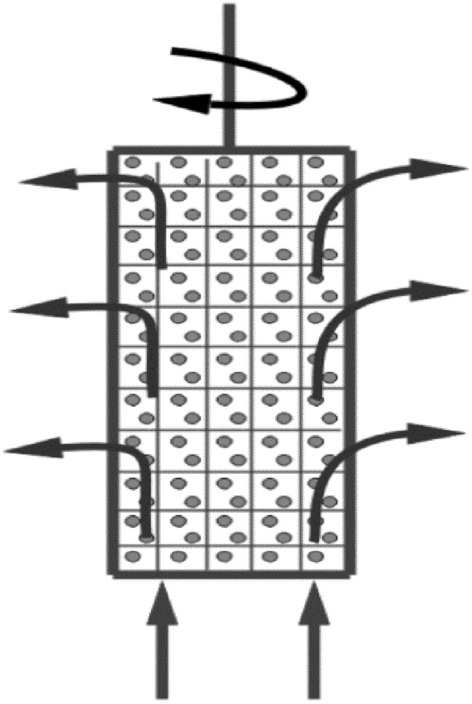
Solution flow pattern during cylinder rotation.

Previous studies explained the increase in mass transfer coefficient with cylinder rotational speed by the fact that cylinder rotation induces an upward axial flow through the porous bed^17,24–26^. The axial flow then moves radially from inside the rotating bed to the outer solution. During this journey of the solution inside the basket, Fig. 4, it enriches the bed with fresh solution and decreases the diffusion layer thickness around each particle, with a consequent increase in the mass transfer coefficient according to Eq. (7)^16^. In addition, it is well known that rotating cylinders are characterized by the formation of a high degree of turbulence at their outer rotating basket surface, with a consequent increase in the rate of (Cu^2+^, Fe^2+^ and  SO_4_^2−^) removal^27^ (Fig. 4).

7 $$K= \frac{D}{\updelta }$$

Previous mass transfer studies showed that film diffusion is the rate-determining step, which is consistent with the present finding^16^.

The obtained data were treated mathematically using dimensionless groups Re, Sh, Sc, and an additional dimensionless factor (L/d) was added to account for the effect of the aspect ratio of the reactor on the rate of mass transfer, where l is the resin height inside the cylindrical basket and d is the diameter of the cylinder.

Re, Sh, and Sc are defined as:

8 $$Re = \frac{{\uprho {\text{Nd}}^{2} }}{\upmu }$$

9 $$Sh= \frac{Kd}{D}$$

10 $$Sc = \frac{\upmu }{{\uprho {\text{D}}}}$$

where ρ is the solution density, N is the cylinder rotation speed, d is the cylinder diameter, µ is the solution dynamic viscosity and D is the mass transfer diffusion coefficient.

**Figure 5:**
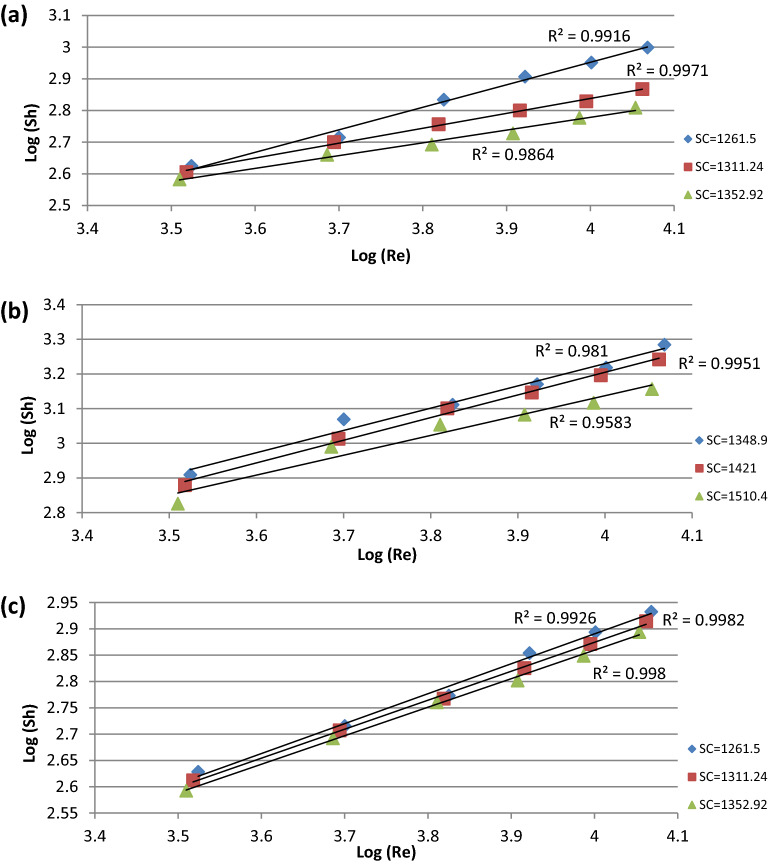
Relation between Sh and Re at different Sc number for (**a**) Cu^2+^ ion, (**b**) Fe^2+^ ion, (**c**) SO_4_^2−^ ion.

The data presented in Fig. 5a–c fit the following equations:

11 $${\text{For}}\;{\text{Cu}}^{2 + } \quad \quad Sh\,\alpha {\text{Re}}^{0.528}$$

12 $${\text{For}}\;{\text{Fe}}^{2 + } \quad \quad Sh\,\alpha {\text{Re}}^{0.622}$$

13 $${\text{For}}\;SO_{4}^{2 - } \quad \quad Sh\,\alpha {\text{Re}}^{0.553}$$

The exponent 0.528 from Eq. (11) is in agreement with the previously obtained values of 0.523 which was obtained by Hagag et al.^16^.

**Figure 6:**
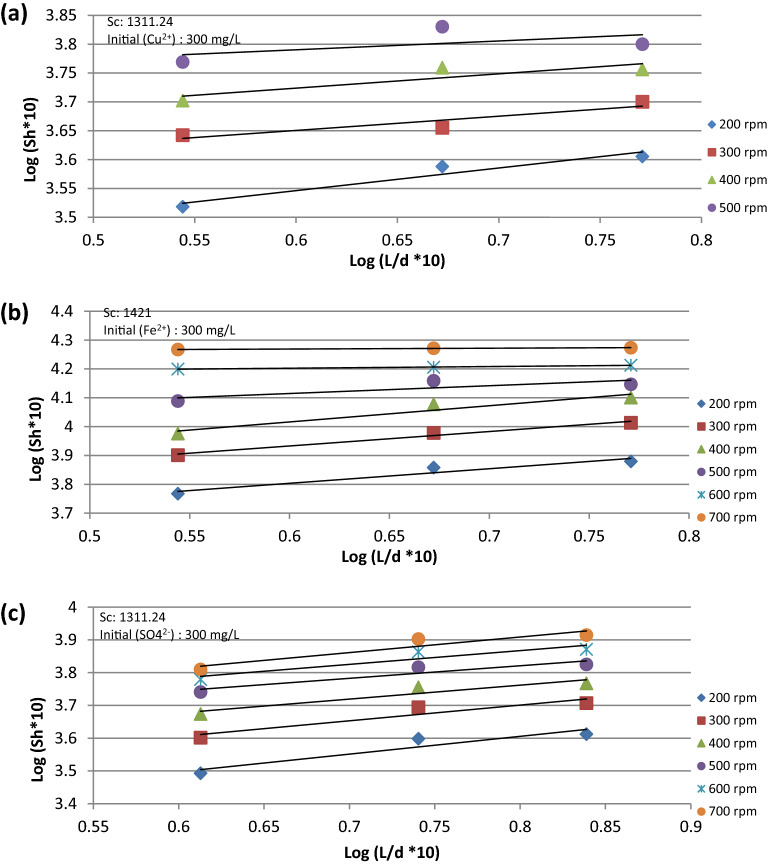
Relation between Sh and L/d at different rpm for (**a**) Cu^2+^ ion, (**b**) Fe^2+^ ion, (**c**) SO_4_^2−^ ion.

The data presented in Fig. 6a–c fit the following equations:

14 $${\text{For}}\;{\text{Cu}}^{2 + } \quad \quad Sh\,\alpha \left( \frac{L}{d} \right)^{0.259}$$

15 $${\text{For}}\;{\text{Fe}}^{2 + } \quad \quad Sh\,\alpha \left( \frac{L}{d} \right)^{0.32}$$

16 $${\text{For}}\;{\text{SO}}_{4}^{2 - } \quad \quad Sh\,\alpha \left( \frac{L}{d} \right)^{0.454}$$

**Figure 7:**
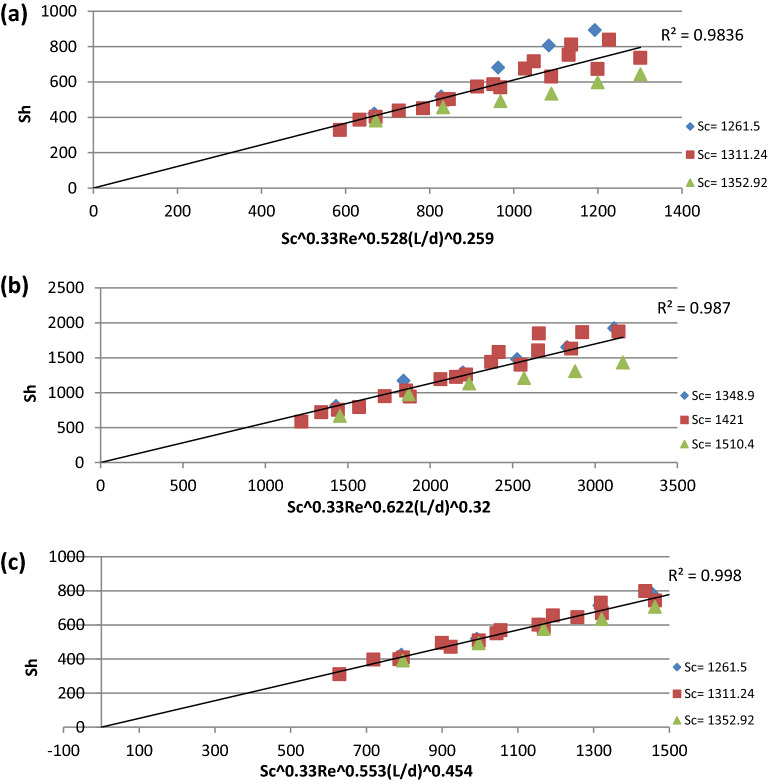
Overall mass transfer correlation for (**a**) Cu^2+^ ion, (**b**) Fe^2+^ ion, (**c**) SO_4_^2−^ ion.

Equations (11, 14) were used in Fig. 7a to obtain an overall mass transfer correlation for Cu^2+^ ion removal as shown in Eq. (17) with an average deviation of ± 9.1% and standard deviation of ± 13.2% under the conditions of 1261.5 < Sc < 1352.9, 3233.94 < Re < 11,697.4, 0.59 < L/d <0.35

17 $$Sh = 0.6114Sc^{{0.33}} \text{Re} ^{{0.528}} \left( {\frac{L}{d}} \right)^{{0.259}}$$

Equations (12, 15) were used in Fig. 7b to obtain an overall mass transfer correlation for Fe^2+^ ion removal as shown in Eq. (18) with an average deviation of ±8.58% and standard deviation of ±11.8% under the conditions of 1348.9 < Sc < 1510.4, 3234.15 < Re < 11,698.53, 0.59 < L/d < 0.35

18 $$Sh = 0.566Sc^{{0.33}} \text{Re} ^{{0.622}} \left( {\frac{L}{d}} \right)^{{0.32}}$$

Equations (13, 16) were used in Fig. 7c to obtain an overall mass transfer correlation for SO_4_^2−^ ion removal as shown in Eq. (19) with an average deviation of ± 3.72% and standard deviation of ± 4.5% under the conditions of 1261.5 < Sc < 1352.92, 3233.9 < Re < 11,697.4, 0.69 < L/d < 0.41

19 $$Sh = 0.5188Sc^{{0.33}} \text{Re} ^{{0.553}} \left( {\frac{L}{d}} \right)^{{0.454}}$$

### Factors affecting the % removal

#### Initial ion concentration

**Figure 8:**
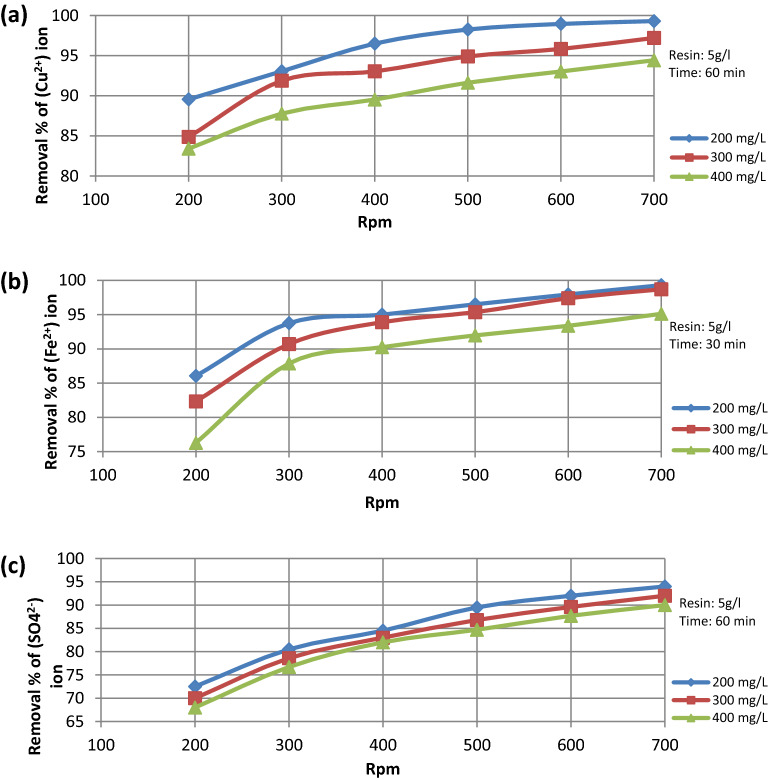
%Removal versus rotational speed at different initial ion concentration for (**a**) Cu^2+^ ion, (**b**) Fe^2+^ ion, (**c**) SO_4_^2−^ ion.

The data presented in Fig. 8a–c show that, as the initial ion concentration increases from 200 to 400 mg/L, the percentage removal decreases. This could be explained by the fact that the amount of resin is constant, thus the same amount of active sites are available for the exchange process, which will be fully occupied and any further ions will remain in the solution. Also, it could be explained by the decrease in diffusion coefficient with increasing the initial ion concentration, which is attributed to the increase in interionic attraction between ions in the solution and the increase in the viscosity of solution^17^.

#### Contact time

**Figure 9:**
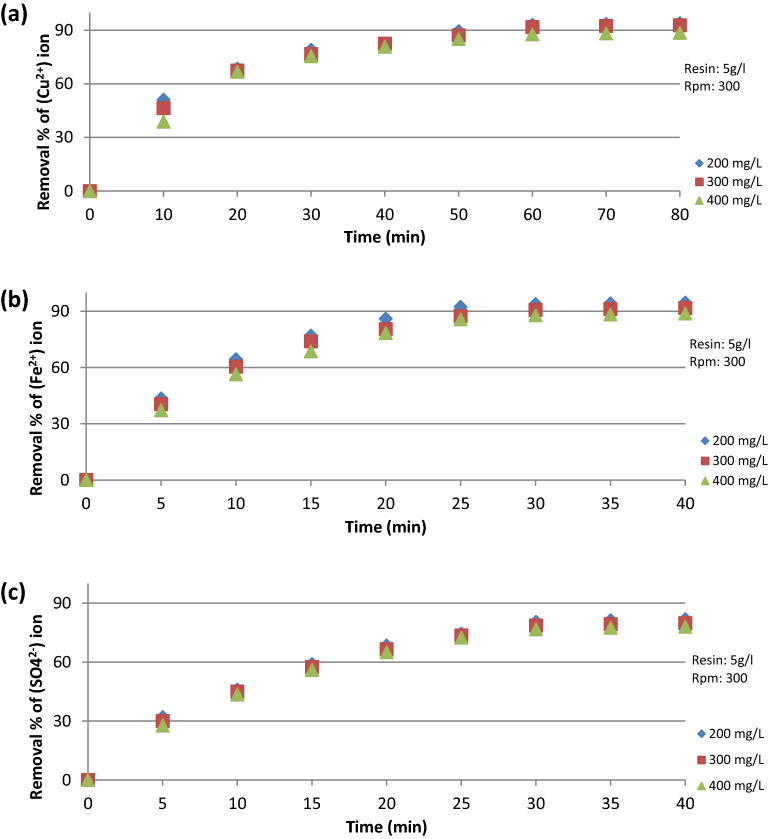
% Removal VS contact time at different initial ion concentration for (**a**) Cu^2+^ ion, (**b**) Fe^2+^ ion, (**c**) SO_4_^2−^ ion.

**Figure 10:**
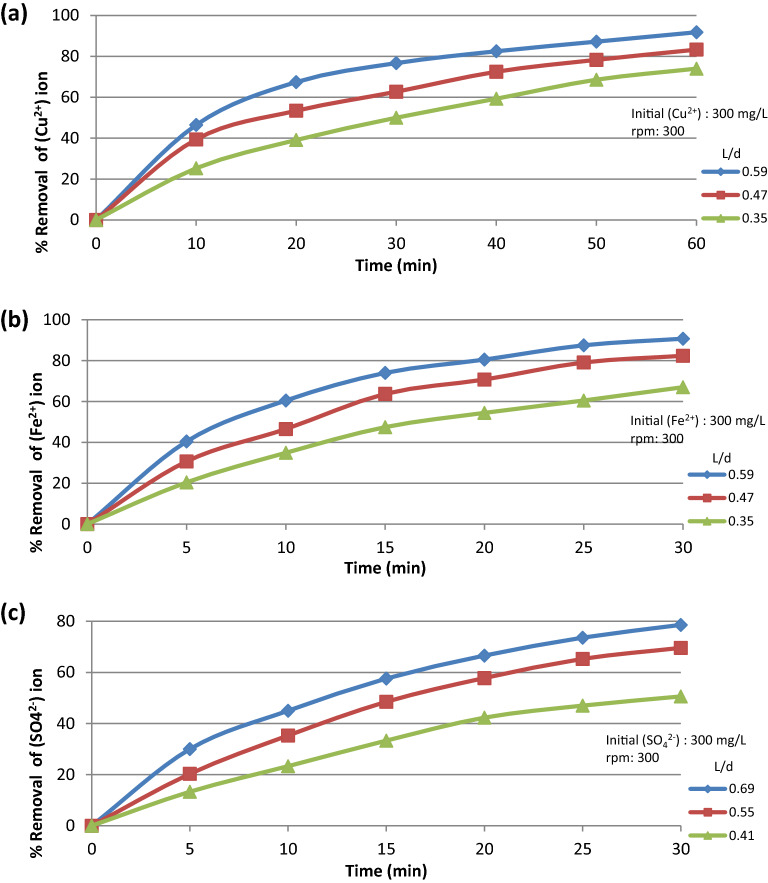
% Removal VS contact time at different L/d ratio for (**a**) Cu^2+^ ion, (**b**) Fe^2+^ ion, (**c**) SO_4_^2−^ ion.

The data presented in Fig. 9a–c show the relation between the percentage removal and the contact time under different initial ion concentrations. It was found that % removal increases as the contact time increases, reaching approximately the equilibrium concentration of (Cu^2+^) after 60 min and of (Fe^2+^, SO_4_^2−^) after 30 min. Figure 10a–c also show that the % removal increases as the contact time increases under different (L/d) ratio.

#### L/d ratio

The increase in K and the % pollutant (Cu^2+^, Fe^2+^, SO_4_^2−^) removal with increasing L/d as shown in Fig. 10a–c may be explained in the light of the following two opposing effects:

1. As L increases, the solution flowing through the bed Fig. 4 becomes depleted in the pollutant. As a result of the decrease in pollutant concentration with bed height, the driving force for the reaction decreases with a consequent decrease in the mass transfer coefficient according to Eq. (2)
2. On the other hand, as the depleted solution arises in the rotating bed, it meets fresh (unexhausted) resin particles with many active centers, which react with the residual ions with a higher degree of removal than the lower partially or fully exhausted resin particles. The increase in K and % pollutant removal with increasing L under the present range of conditions suggests that the second enhancing effect (2) is predominating over the first retarding effect (1). The relatively weak retarding effect arising from pollutant depletion in the upper zones may be ascribed to the partial replenishment of the upper zone by pollutant ions which diffuse from the turbulent outside solution to the upper zone of the rotating basket by virtue of the concentration gradient between the inner solution and the outer turbulent solution of the rotating cylinder basket^27^.

### Ion exchange equilibrium

Equilibrium data is an essential requirement for designing an adsorption system. Adsorption equilibrium is related to temperature, thus at a given temperature, this equilibrium is called an adsorption isotherm. It is used to obtain the maximum possible capacity of the sorbent, where the model which gives the best fit for the obtained experimental results is the best adsorption isotherm to represent those results. Adsorption capacity (q) at equilibrium was obtained from Eq. (20)^16,17,23^.

20 $${q}_{e}= \frac{\left({C}_{o}-{C}_{e}\right)V}{W}$$

where q_e_ is the adsorption capacity after reaching equilibrium (mg/g), C_0_ is the initial concentration in solution (mg/L), C_e_ is the equilibrium concentration in solution (mg/L), V is the used solution volume (L) and W is the dry resin mass (g), knowing that equilibrium time was set to be 60 min for Cu^2+^ ion removal, and 30 min for both Fe^2+^ and SO_4_^2−^ after plotting saturation curves during experimental work.

#### Langmuir isotherm

Langmuir assumed the following points to build up his isotherm^17,23,24^:

- Finite number of active sites arrayed onto the adsorbent surface.
- All active sites show equal adsorption affinity (Homogeneously).
- Only monolayer (single) adsorption takes place.
- Almost negligible interaction occurs between adsorbed molecules.

Langmuir isotherm linear form equation is as follows:

21 $$\frac{{C}_{e}}{{q}_{e}}= \frac{1}{{K}_{L}{Q}_{m}}+ \frac{{C}_{e}}{{Q}_{m}}$$

where K_L_ is the adsorption constant for Langmuir (L/mg), Q_m_ is the maximum theoretical adsorption capacity (mg/g).

**Figure 11:**
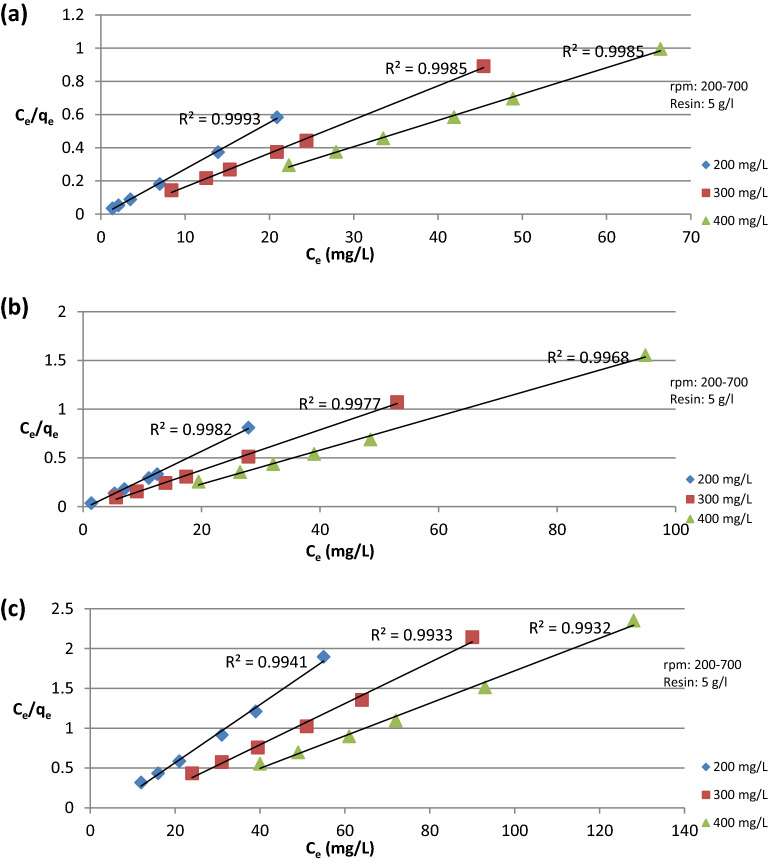
Langmuir adsorption isotherm at different initial ion concentration for (**a**) Cu^2+^ ion, (**b**) Fe^2+^ ion, (**c**) SO_4_^2−^ ion.

The data presented in Fig. 11a–c show the Langmuir adsorption isotherm for different initial ion concentrations, plotting C_e_/q_e_ versus C_e_, from the graphs K_L_, Q_m_ R^2^ of each line were obtained as shown in Table 2.

**Table 2:** Langmuir adsorption isotherm.

Ion	Initial conc (mg/L)	K_L_ (L/mg)	Q_m_ (mg/g)	R^2^
Cu^2+^	200	− 3.255	35.714	0.9993
	300	− 0.51	49.261	0.9985
	400	− 0.227	62.893	0.9985
Fe^2+^	200	− 1.347	33.898	0.9982
	300	− 0.516	48.309	0.9977
	400	− 0.148	57.471	0.9968
SO_4_^2−^	200	− 0.223	27.397	0.9941
	300	− 0.107	38.759	0.9933
	400	− 0.063	49.019	0.9932

#### Freundlich isotherm

This adsorption isotherm is based on the assumption of, a heterogonous surface of an exponential distribution of the adsorption heat over the surface^9,15^.

Freundlich linear form equation is as follows

22 $$Log \,{q}_{e}=Log \,{K}_{F}+ \frac{1}{{\mathrm{n}}_{F}} Log \,{C}_{e}$$

where both K_F_ (L/g) and 1/n_F_ are the adsorption constants for Freundlich.

**Figure 12:**
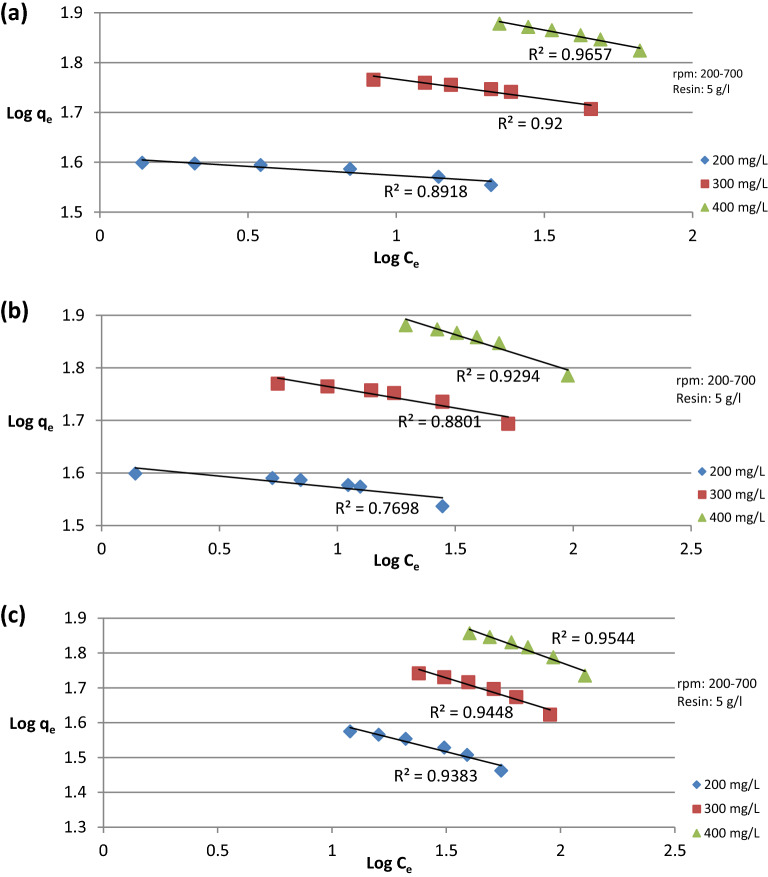
Freundlich adsorption isotherm at different initial ion concentration for (**a**) Cu^2+^ ion, (**b**) Fe^2+^ ion, (**c**) SO_4_^2−^ ion.

The data presented in Fig. 12a–c show the Freundlich adsorption isotherm at different initial ion concentrations, plotting Log q_e_ versus Log C_e_, where from the graph K_F_, 1/n_F_ and R^2^ were obtained as shown in Table 3.

**Table 3:** Freundlich adsorption isotherm.

Ion	Initial conc (mg/L)	K_F_	1/n_F_	R^2^
Cu^2+^	200	40.719	− 0.0363	0.8918
	300	70.194	− 0.0795	0.92
	400	108.043	− 0.1122	0.9657
Fe^2+^	200	41.295	− 0.0436	0.7698
	300	68.754	− 0.0758	0.8801
	400	118.631	− 0.1407	0.9294
SO_4_^2−^	200	58.076	− 0.164	0.9383
	300	107.547	− 0.2019	0.9448
	400	176.97	− 0.2372	0.9544

The obtained R^2^ values in Tables 2, and 3 show that Langmuir adsorption isotherm gives the best fitting for the data obtained from experimental work. The negative values of K_L_ and 1/n_f_ indicate that the adsorption process complies with previous results and confirms that the process won’t be efficient with high adsorbate concentrations^16^.

### Regeneration

Since the cost of ion exchange is a major cost item in the process of wastewater remediation by ion exchange, we decided to examine the efficiency of regenerated resin to make sure that the resin can be used many times without significant loss of efficiency, thus saving on the operating costs of the process.

**Figure 13:**
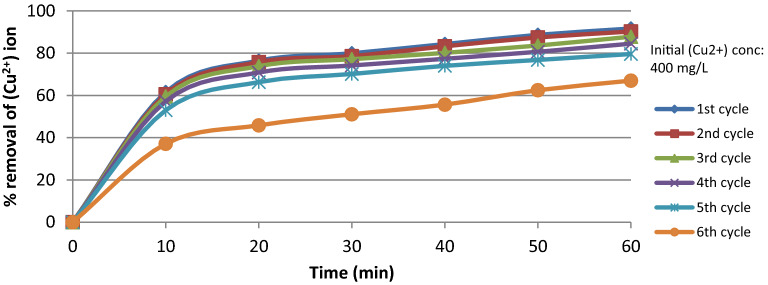
% Removal of (Cu^2+^) versus contact time for adsorption–desorption cycles.

The used strong acid cation (TRILITE MC-10H) reliability was tested by reusing the same amount of resin several cycles, each cycle is meant by the adsorption–desorption reaction. After each adsorption process, the used amount of resin was regenerated using 0.1 M HCl. The data presented in Fig. 13 show the relation between % removal of (Cu^2+^) ion and reaction time at constant 400 mg/L initial (Cu^2+^) concentration and 500 rpm for several cycles for the resin, the graph revealed that the resin showed almost a constant % removal of (Cu^2+^) ion for the first 4 cycles and a slight decrease for the 5th cycle, while a significant decrease for the 6th cycle. This proves that (TRILITE MC-10H) showed great reliability for the (Cu^2+^) ion removal even after using the resin several times.

## Conclusion

The present study has revealed that the rotating ion exchange basket reactor is a highly promising reactor in ion removal from wastewater. Examination of the reactor in the separate removal of (Cu^2+^, Fe^2+^ and SO_4_^2−^) at a rotating fixed bed of ion exchange resin has produced the following results for the conditions: initial (Cu^2+^, Fe^2+^ and SO_4_^2−^) concentration range 200–400 mg/L, pH 5, constant temperature of 25 ± 1 °C, (L/d) ratio range 0.35–0.69 and basket rotating speed range 200–700 rpm.

(1) The system ion exchange process is a first-order diffusion-controlled reaction.

(2) Dimensionless treatment for mass transfer data has yielded the following correlations for (Cu^2+^, Fe^2+^ and SO_4_^2−^) respectively: Eqs. (17)–(19).

These equations can be used in practice to scale up (design) and operate the present reactor.

(3) (TRILITE MC-10H) resin showed great performance to adsorb (Cu^2+^) ion, even after several adsorption–desorption cycles using HCl for regeneration which reflects the magnificent efficiency of the resin particles against heavy duty.

(4) The system showed a higher (Cu^2+^, Fe^2+^ and SO_4_^2−^) ion removal efficiency with increasing basket rotating speed thanks to the flow pattern of axial (inlet)-radial (outlet) which allowed a better circulation for the solution on the resin particles, thus an increased rate of mass transfer.

(5) Equilibrium data studies showed that Langmuir adsorption isotherm gives a better fitting for the obtained experimental data than Freundlich isotherm.

## Data Availability

The datasets generated and analyzed during the study are available from the corresponding author upon request.

## List of symbols

a: Constant
K: Rate constant (cm/s)
V: Solution volume (cm^3^)
W: Resin mass (g)
C0: Initial ion concentration (mg/L)
C_e_: Ion concentration at equilibrium (mg/L)
C: Ion concentration at a given time (mg/L)
A: Resin particle surface area (cm^2^)
d_p_: Resin particle average diameter (cm)
ρ_r_: Resin density (g/cm^3^)
D: Mass transfer diffusion coefficient (cm^2^/s)
t: Time (s)
L: Resin height inside the cylinder basket (cm)
N: Rotation speed (rpm)
δ: Diffusion layer thickness (cm)
µ: Dynamic viscosity of the solution (g/cm. s)
d: Cylinder basket diameter (cm)
ρ: Solution density (g/cm^3^)
q_e_: Adsorption capacity after reaching equilibrium (mg/g)
Q_m_: Maximum adsorption capacity (mg/g)
K_L_: Adsorption constant for Langmuir (L/mg)
K_F_: Coefficient of Freundlich isotherm (L/g)
n_F_: Freundlich isotherm constant
Re: Reynold number (dimensionless)
Sh: Sherwood number (dimensionless)
Sc: Schmidt number (dimensionless)
